# Outcomes of a Routine Screening Protocol to Prevent *Metamycoplasma* and *Ureaplasma* Infection in Lung Transplant Recipients

**DOI:** 10.1111/tid.70221

**Published:** 2026-05-05

**Authors:** Lalithaa Thirunavukarasu Murugan, Anika Sasidharan Nair, Marie M. Budev, Maureen Converse, Nabin K. Shrestha, Christine E. Koval

**Affiliations:** ^1^ Division of Infectious Diseases University of Utah Health Salt Lake City Utah USA; ^2^ Department of Pulmonary Medicine Cleveland Clinic Cleveland Ohio USA; ^3^ Department of Pharmacy MedStar Washington Hospital Center Washington, DC USA; ^4^ Department of Infectious Diseases Cleveland Clinic Cleveland Ohio USA

**Keywords:** hyperammonemia, lung transplant, *Metamycoplasma*, Mollicute, *Mycoplasma*, screening, transplant infection, *Ureaplasma*

## Abstract

**Background:**

*Metamycoplasma hominis* and *Ureaplasma* species (Mollicutes) can cause clinically significant Mollicute infections (csMI) early after lung transplantation (LT). Mollicute infection is associated with hyperammonemia syndrome (HS), a complication resulting in cerebral edema and death. We sought to describe the results before and after a screening protocol to detect and preemptively treat Mollicutes early after LT.

**Methods:**

We retrospectively reviewed LT data at our institution before (January 1, 2020–September 4, 2022) and after (September 5, 2022–August 31, 2023) a screening protocol to detect Mollicutes by PCR on bronchoalveolar lavage within 24 h post‐LT and serum ammonia monitoring every 48 h. Antibiotics were initiated for ammonia > 60 µmol/L or if PCR+. We assessed risk factors and recipient outcomes for Mollicute PCR+ and PCR−. We compared the incidence of HS and csMI in the protocol and pre‐protocol groups.

**Results:**

Screening detected Mollicutes in 24/125 (19.2%). All were treated ≥ 14 days. On multivariable analysis, donor age ≤ 40 years was associated with PCR+ (OR: 4.28, 95% CI: 1.65–12.13, *p* = 0.004). There were no differences in 6‐month mortality (8.3% vs. 7.9%, *p* = 0.61), 3‐month rejection (46% vs. 37%, *p* = 0.19), or mean ICU length of stay (35 days [SD = 29] vs. 42 days [SD = 54], *p* = 0.14) between PCR+ and PCR−. csMI occurred in 1/125 (0.8%) in the protocol group with no HS. Pre‐protocol, 15/1373 (1.09%) developed csMI or HS with two deaths from HS.

**Conclusions:**

Mollicute detection by PCR screened early post‐LT was inversely associated with donor age. Preemptive treatment for Mollicute detection and elevated ammonia in the early posttransplant setting may prevent serious donor‐derived infection.

## Graphical abstract

This study analyzed outcomes of an early Mollicute (*Mycoplasma hominis* and *Ureaplasma* spp.) screening protocol using BAL PCR with preemptive treatment and ammonia monitoring in lung transplant recipients. This appears to be a practical and reasonably effective approach for preventing early Mollicute infection and hyperammonemia syndrome.

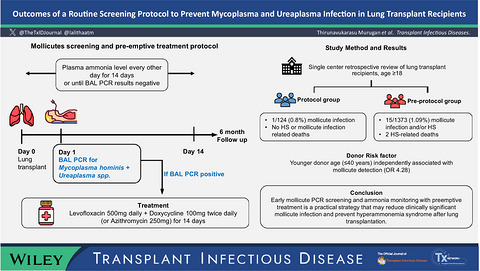

**Abbreviations**
ATG: antithymocyte globulin
BAL: bronchoalveolar lavage
CF: cystic fibrosis
CI: confidence interval
CMV: cytomegalovirus
COPD: chronic obstructive pulmonary disease
csMI: clinically significant Mollicute infection
DCD: donation after circulatory death
HBV: hepatitis B virus
HCV: hepatitis C virus
HIV: human immunodeficiency virus
HS: hyperammonemia syndrome
ICU: intensive care unit
IPF: idiopathic pulmonary fibrosis
IQR: interquartile range
LOS: length of stay
LT: lung transplantation
LTR: lung transplant recipient
MH: *Metamycoplasma (Mycoplasma) hominis*
OR: odds ratio
PCR: polymerase chain reaction
PGD: primary graft dysfunction
PHS: Public Health Service
QTc: corrected QT interval
SD: standard deviation
UNOS: United Network for Organ Sharing
UP: *Ureaplasma parvum*
UU: *Ureaplasma urealyticum*

## 1 Introduction

Mollicute infection due to *Metamycoplasma hominis* (formerly *Mycoplasma hominis*) and *Ureaplasma* species *(U. urealyticum* and *U. parvum*) can cause early infection following lung transplantation (LT). These organisms lack a cell wall, are difficult to detect in cell culture, and do not respond to the β‐lactam antibiotics commonly directed toward suspected infection in the post‐LT setting [1, 2].

Mollicutes most commonly cause early airway and surgical site infections and are linked to hyperammonemia syndrome (HS), a deadly manifestation of infection from these urease‐splitting organisms. HS is reported in 1%–4% of lung transplant recipients (LTRs) but has a mortality up to 75% [2, 3, 4, 5, 6, 7, 8]. Its onset in the first 2 weeks following transplant indicates early infection and organism growth in the setting of immunosuppression [7].

Early post‐LT Mollicute infection appears to be primarily of donor origin rather than recipient colonization at the time of transplant and has previously been linked to younger donor age and sexual activity [5, 6, 8, 9, 10, 11, 12, 13, 14]. Mollicute detection by culture of donor BAL is more predictive of post‐LT infection than detection by PCR, but may be less feasible due to special specimen handling and growth requirements [12].

Given the potential severity of infection, prevention strategies are of inherent interest but require balancing antibiotic exposure with effective prevention. We employed a preemptive strategy to prevent Mollicute infection and HS in our high‐volume lung transplant program by detecting early Mollicute colonization of lower airways and screening for significant serum ammonia levels. We sought to identify donor and recipient factors associated with the detection of Mollicutes. We further attempted to determine the efficacy and outcomes related to the prevention strategy.

## 2 Methods

This study was approved by the Cleveland Clinic Institutional Review Board with a waiver of informed consent due to its retrospective design.

### 2.1 Study Design

In September 2022, our lung transplant program at the Cleveland Clinic began a protocol to screen recipients for Mollicute detection and early hyperammonemia. The protocol included (1) bronchoalveolar lavage (BAL) within 24 h of LT, with BAL fluid tested for PCR detection of *M. hominis*, *U. urealyticum*, and *U. parvum*, sent to Mayo Clinic Laboratory, Rochester, Minnesota; (2) plasma ammonia levels measured every 48 h until the results of the PCR testing were available; (3) if Mollicutes were detected by PCR, treatment was provided with levofloxacin 500 mg daily and doxycycline 100 mg every 12 h (or azithromycin 250 mg daily) for 14 days; and (4) if ammonia levels were >60 µmol/L prior to PCR result availability, the antibiotic regimen was initiated until PCRs resulted and treatment either continued to or stopped for positive and negative results, respectively.

**FIGURE 1:**
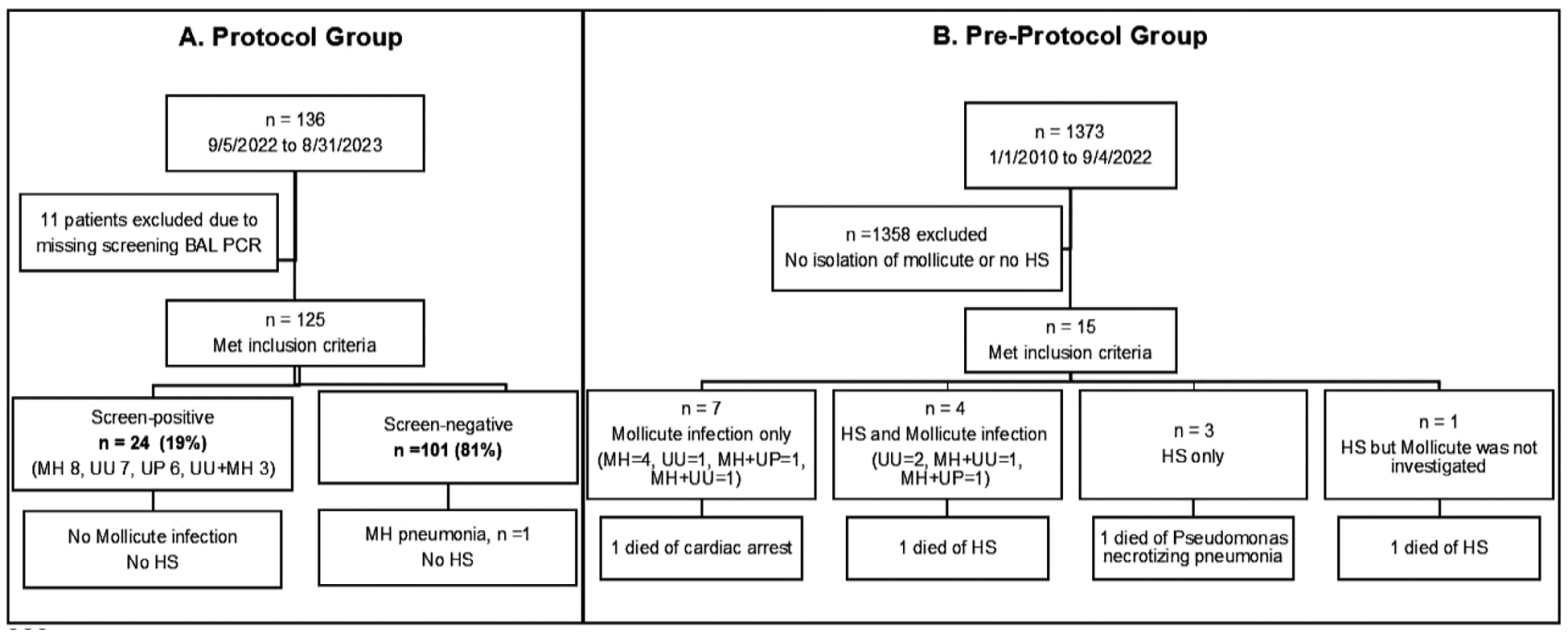
(A) Distribution of Mollicute detection by screening PCR and their clinical outcomes in the protocol group. (B) Distribution of clinically significant Mollicute infection and hyperammonemia syndrome, and their outcomes in the pre‐protocol group. BAL–bronchoalveolar lavage; HS–hyperammonemia syndrome; MH–*Metamycoplasma hominis*; PCR–polymerase chain reaction; UP–*Ureaplasma parvum*; UU–*Ureaplasma urealyticum*.

We retrospectively reviewed records of adult LTRs at Cleveland Clinic Main Campus from January 1, 2010 to August 31, 2023. Patients were divided into two groups: a pre‐protocol group (January 1, 2010–September 4, 2022) and the protocol group (September 5, 2022–August 31, 2023), corresponding to periods before and after the initiation of the Mollicute prevention protocol (see Figure 1).

For the protocol group, we determined the incidence of Mollicute detection by PCR (PCR+). We compared the recipient and donor characteristics and outcomes for those screening PCR+ and PCR−. Donor data were acquired from the United Network of Organ Sharing (UNOS) DonorNet portal, and donors were identified using the UNOS Donor ID and Match ID available in the recipient's medical records. Outcomes included 6‐month survival, 3‐month post‐LT rejection rates, and ICU length of stay during the index transplant admission.

We compared the incidence of HS and csMI in the protocol group to those identified retrospectively through chart review and microbiology data in the pre‐protocol period. HS was defined as a plasma ammonia level >92 µmol/L within 6 months post‐LT, associated with neurological sequelae such as encephalopathy, seizures, and cerebral edema that was not otherwise explained. csMI was defined as the presence of compatible clinical features of infection at any site (including surgical site and anastomotic site infections) with positive microbiological or molecular testing for *M. hominis, U. urealyticum*, or *U. parvum* within 6 months post‐LT.

Although some prior studies have used higher thresholds (typically >200 µmol/L) [4, 10] to define HS, we selected a lower cutoff to improve sensitivity for early detection, given the high reported mortality associated with this condition. This threshold also remains above the institutional upper limit of normal (60 µmol/L) to minimize inclusion of mild post‐LT elevations not typically associated with HS.

Standard perioperative LT antimicrobial prophylaxis during the protocol period included vancomycin plus cefuroxime or piperacillin‐tazobactam for 72 h; antiviral prophylaxis with acyclovir or valganciclovir (based on CMV serostatus) for 12 months; trimethoprim‐sulfamethoxazole prophylaxis indefinitely for pneumocystis; posaconazole for 3 months with inhaled amphotericin B until therapeutic levels were achieved; and oral vancomycin for *Clostridioides difficile* prophylaxis during the peri‐transplant ICU stay. Notably, these regimens lack activity against Mollicutes. Routine surveillance bronchoscopies were performed at 3 and 6 weeks, and at 3, 6, and 9 months post‐LT. Specific Mollicute testing was not routinely included in standard BAL microbiologic evaluation for either surveillance or other clinically indicated bronchoscopies.

### 2.2 Statistical Analysis

Continuous variables are reported as means with standard deviation or median with range. Demographic variables are reported as numbers and percentages. Categorical variables have been compared using a two‐sided Fisher's exact test or a chi‐square test for categorical data with ≥ three categories, a Welch *t*‐test for mean, or a Mann–Whitney *U* test for median. Univariable and multivariable logistic regression were used to assess the association between donor characteristics and screening test positivity. A *p*‐value ≤ 0.05 was considered statistically significant. All analyses were performed using R Statistical Software (v4.3.2).

## 3 Results

### 3.1 Mollicute Detection by PCR

A total of 136 patients underwent LT in the screening protocol period, with 11 excluded due to missed BAL. The median number of days from LT to screening BAL PCR test was 1 day (range: 1–10, IQR: 1–1) and the median BAL PCR test turnaround time was 4 days (range: 2–9, IQR: 4–5). Of 125 screened, 24 (19.2%) patients were PCR+ and 101 (80.8%) were PCR−. Among the PCR+, 8 were *M. hominis*, 7 were *U. urealyticum*, 6 were *U. parvum*, and 3 were positive for both *U. urealyticum and M. hominis* (see Figure 1). Two single lung recipients from the same donor were PCR+ for *U. parvum*.

### 3.2 Risk Factors for Mollicute Detection by Screening

Donor and recipient factors were compared between PCR+ and PCR− groups. There were no differences in recipient characteristics, including age, sex, race, organs transplanted, pretransplant immunosuppression, induction immunosuppression, and underlying diagnosis requiring lung transplant (see Table 1).

**TABLE 1:** Recipient baseline characteristics in the protocol cohort by screening Mollicute PCR result.

	Screen positive *n* = 24	Screen negative *n* = 101	*p* value
Age in years, mean (SD)	58.96 (11.27)	61.50 (8.39)	0.27
Sex, *n* (%)			0.64
Male	17 (71%)	64 (63%)	
Female	7 (29%)	37 (37%)	
Race, *n* (%)		​	0.21
White	24 (100%)	89 (88%)	
Black	0	7 (7%)	
Other	0	5 (5%)	
Lung as only organ transplanted, *n* (%)	24 (100%)	95 (94%)	0.59
First lung transplant, *n* (%)	22 (92%)	99 (98%)	0.16
Bilateral lung transplant, *n* (%)	21 (87.5%)	96 (95%)	0.18
Pretransplant immunosuppression, *n* (%)	8 (33%)	28 (28%)	0.62
Indication for lung transplant, *n* (%)			0.37
IPF	10 (42%)	53 (53%)	
COPD	8 (33%)	21 (21%)	
CF	1 (4%)	1 (1%)	
Others	5 (21%)	26 (26%)	
Induction immunosuppression, *n* (%)			0.98
ATG	9 (37.5%)	37 (37%)	
Basiliximab	1 (4%)	5 (5%)	
No induction	15 (62.5%)	60 (59%)	

Abbreviations: ATG, antithymocyte globulin; CF, cystic fibrosis; COPD, chronic obstructive pulmonary disease; IPF, idiopathic pulmonary fibrosis.

There were no differences in the following donor characteristics (see Table 2): race, cause of death, donation after brain death or circulatory death, time from hospital admission to cross clamp, CMV serostatus, donor region per US Census Bureau's regions, and fulfillment of risk criteria for HIV, HBV, or HCV per 2020 Public Health Service (PHS) guidelines. On univariable analyses, donor age ≤ 40 years (OR: 4.58, 95% CI: 1.80–12.83, *p* = 0.002) and male sex (OR: 2.90, 95% CI: 1.15–8.07, *p* = 0.03) were associated with increased odds of Mollicute detection. Only donor age ≤ 40 years remained significant in multivariable analysis (OR: 4.28, 95% CI: 1.65–12.13, *p* = 0.004).

**TABLE 2:** Donor characteristics associated with recipient screening Mollicute PCR positivity—univariable and multivariable logistic regression analysis.

	Screen‐positive, *n *= 24	Screen‐negative, *n *= 101	Univariable OR (95% CI)	Univariable *p* value	Multivariable OR (95% CI)	Multivariable *p* value
Male sex, *n* (%)	17 (71%)	46 (45.5%)	2.90 (1.15–8.07)	0.030	2.62 (0.99–7.50)	0.06
Age ≤ 40, *n* (%)	17 (71%)	34 (24%)	4.58 (1.79–12.82)	0.002	4.28 (1.65–12.13)	0.004
Time from admission to cross clamp >5 days, *n* (%)	19 (79%)	73 (72%)	1.46 (0.53–4.73)	0.49		
Donation by cardiac death (DCD), *n* (%)	10 (42%)	30 (30%)	1.69 (0.66–4.21)	0.26		
Drug overdose as cause of death, *n* (%)	6 (25%)	20 (20%)	1.92 (0.51–7.47)	0.33		
Positive donor CMV serostatus, *n* (%)	15 (62.5%)	60 (59%)	1.09 (0.44–2.82)	0.85		
Donor from south region, per US Census bureau's region, *n* (%)	7 (29%)	30 (30%)	1.33 (0.34–4.35)	0.67		
Fulfilling at least one of the risk criteria for HIV, HBV, or HCV identified in the 2020 PHS guideline [15], *n* (%)	4 (17%)	13 (13%)	1.35 (0.35–4.31)	0.63		

Abbreviations: CMV, Cytomegalovirus; HBV, Hepatitis B Virus; HCV, Hepatitis C Virus; HIV, Human Immunodeficiency Virus; PHS, Public Health Service.

Data on donor antibiotic exposure were not uniformly available and therefore were not captured in this study. Consequently, we were unable to determine whether donors received Mollicute‐active antibiotics or whether such exposure may have influenced screening PCR results.

### 3.3 Hyperammonemia

Note that, 8 of 136 patients did not receive ammonia monitoring; all were screened and PCR−. Among those tested, the median peak plasma ammonia level was 36 µmol/L (range: 20–119, IQR: 31–45) in PCR+ recipients and 32 µmol/L (range: 10–119, IQR: 27–41) in PCR− recipients, with no significant difference between groups (*p* = 0.8, Mann–Whitney *U* test).

De novo ammonia levels >60 µmol/L were detected in 4 of 24 (16.6%) that were PCR+ and 3 of 101 (3%) that were PCR−. For those with elevated ammonia and PCR+, antimicrobial therapy in response to the PCR results was started on the same day or prior to the detection of elevated ammonia. All subsequent ammonia levels normalized quickly. One of the four patients developed a rapid increase in plasma ammonia levels, peaking at 119 µmol/L, without associated neurological sequelae. This occurred within 24 h prior to detection of *Metamycoplasma* and *Ureaplasma* by PCR on post‐LT Day 7. The patient received targeted therapy for hyperammonemia (lactulose, thiamine, vitamin B6, zinc, selenium, rifaximin, and levocarnitine) along with prompt initiation of Mollicute‐directed antimicrobial therapy. Ammonia levels subsequently normalized within 4 days.

For those with elevated ammonia but PCR−, none received Mollicute‐directed antibiotics. No intervention was done for one patient (with peak ammonia 70), valproate was stopped for another (with peak ammonia 63), and third patient (with peak ammonia 119), who experienced a watershed cerebral infarct in the immediate postoperative setting was treated for hyperammonemia with lactulose, metronidazole, thiamine, vitamin B6, zinc, selenium, rifaximin, buphenyl, and levocarnitine, but had a clear reason for altered cognition.

### 3.4 Antimicrobial Therapy

All screen‐positive patients received preemptive treatment for a median duration of 15 days (range: 14–46, IQR: 14.5–20.5). Treatment regimens included levofloxacin + doxycycline in 21 patients, levofloxacin + azithromycin in 2 patients, and doxycycline alone in 1 patient.

Most patients received 14–15 days of therapy. Duration recorded as 15 days reflects staggered initiation of the two antimicrobial agents on consecutive days rather than simultaneously. A small number received extended courses (up to 46 days) early during protocol implementation, before treatment practices were standardized, when some providers interpreted positive PCR results as deeper infection rather than colonization.

Seventeen of 125 patients were treated with Mollicute‐active antibiotic directed to another organism prior to the screening PCR result. Most were started on the day of or after screening BAL PCR collection, except for four patients who started treatment 3–5 days before BAL collection; all four were screen‐negative (one on ciprofloxacin for *Pseudomonas*, one on levofloxacin for broad coverage, one levofloxacin for *Stenotrophomonas*, one on clarithromycin for *Helicobacter pylori*).

### 3.5 Clinically Significant Mollicute Infection Without HS

One patient in the screen‐negative group developed *M. hominis* pneumonia on post‐LT Day 36. The patient had difficulty weaning from mechanical ventilation and required tracheostomy. Bronchoscopy demonstrated purulent bilateral lower airway secretions and early dehiscence of the left mainstem bronchial anastomosis. Routine BAL bacterial culture grew *M. hominis* (70 000 CFU/mL) within 72 h. Following targeted antimicrobial therapy, airway secretions resolved, and the bronchial anastomosis showed evidence of healing. The patient completed a 15‐day treatment course.

### 3.6 Other Posttransplant Outcomes

Six‐month posttransplant mortality, tracheostomy requirement during index hospitalization for transplant, ICU length of stay during index hospitalization for transplant, 72‐h primary graft dysfunction (PGD) grading, rejection requiring treatment within 90 days post‐LT, were not different between screen‐positive and screen‐negative groups (see Table 3).

**TABLE 3:** Posttransplant recipient outcomes by screening Mollicute PCR results in the protocol group.

	Screen‐positive, *n* = 24	Screen‐negative, *n* = 101	*p* value
Tracheostomy in index admission	16 (67%)	55 (54.5%)	0.36
Mean ICU LOS in days in index admission	35 (SD 29)	42 (SD 54)	0.38
≥1 rejection requiring treatment within 90 days post‐LT	10 (42%)	33 (33%)	0.48
6‐month post‐LT mortality	2 (8%)	8 (8%)	1.0
Mollicute infection in 6 months post‐LT	0	1 (1%)	1.0
HS in 6 months post‐LT	0	0 (0%)	—
T72 PGD	​	​	0.15
Grade 0	7 (29%)	13 (13%)	
Grade 1	12 (50%)	49 (48%)	
Grade 2	1 (4%)	11(11%)	
Grade 3	0 (0%)	12(12%)	
Ungradable/not mentioned	4(17%)	16(16%)	
Median of the longest QTc between Day 15 and Day 20 post‐LT	Checked in 16 of 24 (67%) Median 442 (range 389–524)	Checked in 67 of 101 (66%) Median 435 (range 279–565)	—

We collected the highest QTc values between Days 15 and 20 post‐LT (see Table 3). Medians were similar between groups. However, a formal statistical comparison was not performed due to incomplete data. Other antibiotic‐related adverse events, including *C. difficile* infection and tendon injury, were not systematically assessed.

### 3.7 Impact of Screening Protocol

Before the screening protocol initiation, there were 1373 LTRs, with 15 developing HS and/or Mollicute infection within 6 months post‐LT. Among these 15 patients, 7 had only Mollicute infection, 4 had HS and Mollicute infection, 3 had only HS, and 1 had HS, but Mollicute infection was not investigated. Note that 4 of the 15 (26.6%) patients died within 6‐month post‐LT, with two deaths attributed to HS. In patients who developed HS (*n*  =  8), median peak ammonia was 269.5 µmol/L (range: 114–361, IQR: 179–321) and median number of days from transplant to peak ammonia level was 13 days (range: 7–30, IQR: 9–14.5). In patients with Mollicute infection (*n*  =  11), the median days from transplant to first positive test date was 13 (range: 6–176, IQR: 10–28).

Mollicute infection was diagnosed in 1/125 (0.8%) of the protocol group with no attributable deaths from Mollicutes or HS. In comparison with 15/1373 (1.09%) of the pre‐protocol group, with two deaths attributable to HS.

## 4 Discussion

Mollicute infections caused by *M. hominis* and *Ureaplasma* species are increasingly recognized as serious complications following LT. Their association with HS, a highly fatal condition marked by progressive neurologic deterioration due to elevated serum ammonia, has drawn growing concern. Our findings support existing literature indicating that these infections are donor‐derived, and that systematic screening coupled with preemptive treatment may reduce adverse outcomes.

In our retrospective analysis, 19.2% of LTRs screened by BAL had Mollicutes detected on PCR. This prevalence aligns with previously reported colonization rates ranging from 11% to 26% [5, 6, 10, 12, 14]. Our data further reinforce prior observations that younger donor age is associated with increased risk of Mollicute positivity [5, 8, 10, 13, 14].

Despite a relatively high rate of PCR detection, the incidence of csMI and HS was low following implementation of the screening protocol. Only one of 125 screened recipients developed a csMI, and no cases of HS occurred. In contrast, 15 of 1373 recipients in the pre‐protocol cohort developed csMI or HS, including two HS‐attributed deaths. These findings suggest that protocolized screening combined with preemptive antimicrobial therapy may reduce the incidence and severity of Mollicute‐associated complications after LT.

Interestingly, the single csMI case in the protocol group occurred in a patient who had screened‐negative, highlighting limitations of current diagnostic strategies and the potential for false negative screening results. In our practice, additional testing for Mollicutes beyond the initial screening assay was not routinely performed. Additional testing with Mollicute PCR or culture of BAL, blood, or tissue was pursued only when infection was clinically suspected, and initial evaluations were unrevealing. Despite increasing awareness, Mollicute infections may remain underrecognized, and clinicians should maintain a high index of suspicion for Mollicute infection and HS in the early post‐LT period.

The observed incidence of identified csMI and HS in the pre‐protocol cohort was 0.8% (11 of 1373) and 0.6% (8 of 1373), respectively. However, these rates likely underestimate the true burden of disease, as the pre‐protocol period spanned many years during which awareness of HS and Mollicute infections was limited, and Mollicute diagnosis required deliberate specialized culture, PCR testing, or chance detection on routine cultures.

Our institutional protocol differed from prior approaches in two important ways: first, we incorporated routine serial ammonia monitoring in the early post‐LT period to detect subclinical elevations; second, we could initiate preemptive antimicrobial therapy based on either elevated ammonia (>60 µmol/L) or positive PCR results, rather than awaiting the development of clinical symptoms. However, in no case was antimicrobial therapy initiated in response to elevated ammonia levels alone since the PCR results had resulted on the same day or prior to the ammonia elevations. Nonetheless, this dual approach may provide an added safety margin and facilitate timely intervention.

Our findings also contribute to the ongoing discussion regarding optimal screening methodologies. Prior study demonstrated that Mollicute culture of donor BAL was more predictive of post‐LT infection than PCR detection [12], suggesting that PCR may be overly sensitive and detect colonization, potentially leading to overtreatment in a preemptive model. The choice of diagnostic modality should be guided by institutional resources, assay availability, turnaround times, and laboratory capabilities. Despite a median PCR turnaround time of 4 days at our center, concurrent ammonia monitoring enabled the timely identification of at‐risk patients and the initiation of therapy. Several centers send Mollicute testing directly from the donor lungs prior to transplant [10, 12]. While this clearly characterizes detected organisms as of donor origin, we think that lower airway testing of the recipient lungs early after implantation also reflects material of donor origin. This changes if the post‐LT testing is delayed, for example, in the setting of PGD. However, we do not think that this detracts from the clinical relevance of a later positive result.

Early PCR positivity was presumed to reflect donor‐derived colonization rather than established infection. Our preemptive strategy was therefore designed to identify and treat colonization early, with the goal of preventing progression to csMI and HS. Given the high morbidity and mortality associated with HS, this approach favors early intervention despite the potential for overtreatment, highlighting the need to balance antimicrobial stewardship with prevention of rare but catastrophic outcomes.

Although ammonia monitoring did not independently trigger antimicrobial initiation in this cohort, it may serve as an adjunctive safety mechanism, particularly in settings with delayed PCR turnaround times. In four cases, rising ammonia levels coincided with positive Mollicute PCR results, including one patient whose ammonia increased to 119 µmol/L approximately 7 days post‐LT. These observations suggest that ammonia trends may play a supportive, rather than determinative, role in clinical decision‐making.

We observed no differences in ICU length of stay, tracheostomy rates, early graft rejection, or 6‐month mortality between screen‐positive and screen‐negative recipients. These data suggest that colonization alone does not increase early post‐LT morbidity when effective prophylaxis is provided. It also suggests that the added BAL and preemptive therapies do not affect long‐term outcomes. This finding supports a targeted treatment approach rather than universal prophylaxis, which may unnecessarily increase antimicrobial exposure.

Two recipients who received lungs from the same donor both tested positive for *U. parvum*. This adds to previous studies confirming donor‐derived transmission of Mollicutes, including those employing whole‐genome sequencing to demonstrate genetic concordance between donor and recipient isolates [9, 11].

While some transplant centers have studied universal donor and recipient prophylaxis treatment strategies [10, 16], our data support a targeted approach using PCR and ammonia screening, which balances efficacy with antibiotic stewardship.

Our findings demonstrate that a protocolized approach combining systematic screening with preemptive treatment may reduce infection and prevent serious Mollicute‐associated complications following LT.

This study has several limitations. First, it is a retrospective, single‐center study, which may limit generalizability and introduce the potential for selection and measurement bias. Second, the comparison between the pre‐protocol and protocol periods spans more than a decade, introducing potential temporal bias due to evolving transplant practices, improved diagnostic awareness, and changes in supportive care over time. While restricting the analysis to more recent years could mitigate this effect, doing so would substantially reduce event numbers given the rarity of outcomes. Third, though no HS case was observed in the protocol cohort (0/125), the upper bound of the 95% CI is approximately 2.4%, indicating that a small but nonzero risk cannot be excluded. Fourth, donor antimicrobial exposures were not consistently available and could not be evaluated as a potential confounder. Fifth, we did not systematically assess antibiotic‐related adverse events such as *C. difficile* infection or tendonitis in the protocol group, nor did we evaluate overall surgical site or anastomotic complications across groups. These limitations highlight the need for prospective, multicenter studies to better define optimal screening and treatment strategies.

## 5 Conclusion

In summary, early Mollicute detection using BAL PCR combined with serial ammonia monitoring and preemptive antimicrobial therapy represents a practical prevention strategy that was associated with a low incidence of csMI and no observed cases of HS in this cohort. Although causal conclusions cannot be definitively established given the retrospective design and low event rates, these findings support a targeted detection and treatment approach for Mollicute‐associated complications after LT.
